# Determinants of Long‐Term Benefit From High Dose Melphalan With Autologous Stem Cell Transplant in AL Amyloidosis

**DOI:** 10.1002/ajh.70371

**Published:** 2026-06-07

**Authors:** Maximilian J. Steinhardt, Ute Hegenbart, Tamer Hellou, Sara Oubari, Murielle Roussel, Rahel Schwotzer, Francis Buadi, David Dingli, Moritz Binder, Taxiarchis Kourelis, Saurabh S. Zanwar, Max J. Rieger, Caroline Morbach, Vladimir Cejka, Bernhard Gerber, Stefan Störk, Hermann Einsele, Shaji Kumar, Morie Gertz, Alexander Carpinteiro, K. Martin Kortum, Eli Muchtar, Angela Dispenzieri, Stefan Schönland

**Affiliations:** ^1^ Department of Hematology Mayo Clinic Rochester Minnesota USA; ^2^ Department of Internal Medicine II University Hospital of Würzburg Würzburg Germany; ^3^ Interdisciplinary Amyloidosis Center University Hospital Würzburg Würzburg Germany; ^4^ Department of Internal Medicine V, Amyloidosis Center Heidelberg University Hospital Heidelberg Germany; ^5^ Department of Hematology and Stem Cell Transplantation University Hospital Essen Essen Germany; ^6^ West German Amyloidosis Center University Hospital Essen Essen Germany; ^7^ Department of Hematology CHU Limoges Limoges France; ^8^ Department of Medical Oncology and Haematology University Hospital of Zurich Zürich Switzerland; ^9^ Università Della Svizzera Italiana Lugano Switzerland; ^10^ Department of Hematology Sheba Medical Center Tel Hashomer Israel

## Abstract

High dose melphalan (HDM) with autologous stem cell transplant is an established treatment for systemic light chain amyloidosis, but its incremental benefit in the era of effective standard intensity therapy is unknown. We retrospectively analyzed 475 transplant‐eligible patients who completed standard intensity treatment with or without HDM within 12 months at six centers in Europe and the United States between 2010 and 2024 to evaluate outcomes by baseline risk factors, hematologic response, and receipt of HDM. Death was an equally competing risk after the 12‐month landmark chosen to address early non‐proportional hazards and to ensure completion of first‐line therapy. BMPC > 20% was associated with inferior outcomes (HR 1.7), and gain/amp 1q with shorter TTP (HR 2.15), whereas other high‐risk FISH abnormalities were not. In multivariable models, VGPR/CR after standard intensity therapy was associated with longer TTP (HR 0.49/0.29), while gain/amp 1q remained independently associated with shorter TTP (HR 2.18). Receipt of daratumumab and HDM were associated with longer TTP (HR 0.44/0.61). HDM benefited patients who had not achieved CR after standard intensity therapy (mTTP 21.0 vs. 5.7 months without HDM), whereas patients already in CR did not benefit. Higher BMPC at diagnosis was associated with earlier progression in patients not receiving HDM (HR 1.9 for 5%–20% and 4.09 for > 20%), a risk attenuated in the HDM cohort. Gain/amp 1q remained associated with shorter TTP regardless of HDM exposure. These findings support a selective role for HDM, primarily benefiting patients with residual disease after standard intensity therapy or higher BMPC burden at diagnosis.

## 1 Introduction

Systemic light chain amyloidosis (AL) is a severe disease caused by organ deposition of misfolded free light chains (FLC) most commonly produced by clonal plasma cells. Progressive aggregation leads to disruption of organ function and ultimately organ failure.

High dose melphalan (HDM) with autologous stem cell transplant is derived from multiple myeloma (MM) therapy and is considered a cost‐effective and highly active therapy for AL [1, 2]. Treatment with HDM has resulted in deep hematologic responses with long progression‐free survival (PFS) and overall survival (OS) [3], especially when achieving hematologic complete remission (CR) [4, 5]. It is considered one of the most effective treatments available for eligible, that is, very fit, patients [6]. Especially in AL, however, it is associated with significant toxicity. Due to higher mortality compared to other indications, its benefit is dependent on careful patient selection, and the definition of transplant eligibility is complex [2, 7, 8, 9]. Furthermore, the positive impact on PFS and OS by HDM in retrospective analyses may be in part due to immortal time bias, as patients must survive and remain progression‐free to receive HDM [10], and a selection bias with difference in outcomes due to different therapeutic options, patient selection, center preferences, and supportive therapy [6, 11, 12].

The introduction of daratumumab‐based induction regimens has further challenged the historical paradigm of upfront HDM for all eligible patients because these regimens often achieve rapid and deep hematologic responses. Moreover, they have shown good tolerability in first and second line [13, 14]. Because of this, HDM has been used less frequently and has been reserved for unsatisfactory response, usually defined as less than VGPR or lack of organ response; some patients may not require HDM at all [15].

AL patients are heterogeneous not only in organ involvement and AL‐associated morbidity but also due to the underlying plasma cell phenotype. These have been categorized by [1] bone marrow plasma cell (BMPC) infiltration [2], cytogenetic phenotype, usually assessed via fluorescence in situ hybridization (FISH), and [3] laboratory parameters derived from AL and MM studies, such as FLC levels [16], beta‐2 microglobulin [17], and LDH [18].

While it is well established that higher BMPC infiltration is a negative risk factor for outcomes [5, 19], the impact of MM‐derived FISH abnormalities in outcomes in AL is less clear and may depend on context [20]. It has been assessed in several studies, with gain/amp1q [21, 22, 23] standing out as a significant risk factor. Interestingly, t(11;14) has been described as a risk factor with inferior response to bortezomib [24] that can be overcome by HDM [25], whereas the impact of del17p appears to be diminished compared to MM [26]. Overall, the definite incremental benefit of HDM after induction therapy in the light of specific risk factors is unknown to date.

To address this knowledge gap, we analyzed a large multi‐center cohort of transplant‐eligible patients. We aimed to [1] assess outcomes depending on plasma‐cell derived risk factors [2]; stratify by treatment decisions for or against HDM made by clinicians; and [3] evaluate the role of HDM in the light of the underlying plasma cell disease.

## 2 Methods

### 2.1 Ethics Approval and Consent to Participate

This study involved analysis of existing clinical data and was approved by Medizinische Ethikkommission an der Julius‐Maximilians‐Universität Würzburg (202699dvhd) and the Institutional Review Board (24–009382). All proceedings are in accordance with the Declaration of Helsinki and other relevant guidelines and regulations. No identifiable images or personal data of participants are included in this publication.

### 2.2 Study Cohort

We retrospectively analyzed data from transplant‐eligible patients as defined by EHA‐ISA criteria [27, 28] with AL from six academic centers in Europe and the United States from 2010 to 2024. Patients were eligible if they had systemic AL defined by biopsy proof and the presence of a corresponding monoclonal plasma cell disorder. Patients receiving HDM without prior induction, as salvage due to lack of response (≤ PR), after more than 12 months, after more than one line of therapy or after induction with less than a triplet were excluded from the analysis. Patients not receiving HDM had to receive limited duration therapy containing at least a triplet combination < 12 months. Single‐agent maintenance was permitted in both groups. Transplant eligibility criteria were applied retrospectively based on recorded clinical parameters at treatment initiation. In central adjudication, they also had to fulfill transplant eligibility criteria as formulated previously [6, 27, 28]: Age ≤ 70 years, ECOG Performance Score ≤ 2, systolic blood pressure ≥ 90 mmHg, TnT < 0.06 ng/mL (or hs‐TnT < 75 ng/L), CrCl ≥ 30 mL/min (unless on chronic dialysis), and NYHA Class < III. Very low dFLC patients (dFLC < 20 mg/L) were excluded from the analysis (Figure S1).

### 2.3 Data Assessment

Data extraction from the electronic medical record were performed. Baseline variables captured included demographic, laboratory parameters, BMPC infiltration, and cytogenetics. We also assessed duration, dosage, type, and number of substances used in first‐line therapy. Outcome measures included hematologic response, survival, time to progression (TTP), and time to next treatment. Because the cohort consisted of a fit, transplant‐eligible population with expected low early mortality, TTP (progression as defined by the international society of amyloidosis (ISA [29]) or initiation of second‐line treatment before fulfillment of formal progression criteria treated as progression events, and death treated as a censor event) was selected as the primary endpoint. Landmark analyses of patients alive and progression‐free at 12 months after initiation of therapy were performed to address early non‐proportional hazards, avoid immortal time bias related to delayed HDM administration and to ensure completion of first‐line therapy and response assessment. Sensitivity analyses for other timepoints and outcome interaction analyses were also performed. Organ involvement was assessed as proposed by ISA guidelines [30]. Standard intensity treatment is referred to as induction if patients proceeded to HDM. Response after HDM or standard intensity regimen was assessed at the 12‐month landmark.

Serologic response was categorized as CR, very good partial response (VGPR), partial response (PR), stable disease (SD), or progressive disease (PD) according to ISA 2012 criteria [29]. High‐risk FISH abnormalities were chosen according to the multiple myeloma R2 ISS criteria [31].

### 2.4 Statistical Analysis

Differences between groups were analyzed using Chi‐squared test for categorical variables and Wilcoxon rank‐sum for continuous variables. Kaplan–Meier survival curves were compared using the log‐rank test for overall differences in survival. Continuous data are described as median and quartiles. Univariable and multivariable Cox models were fitted to evaluate the influence of possible prognostic factors on TTP and OS. To illustrate the results of the Cox models, hazard ratios were calculated. No data imputation was performed. Cumulative OS and progression events were compared between groups using Fine and Gray's competing risk regression. *p* values less than 0.05 were considered significant. JMP18 (SAS) was used for statistical analysis.

## 3 Results

### 3.1 Study Population

Of 569 transplant‐eligible AL patients identified by EHA‐ISA criteria between 2010 and 2024 without concurrent MM, 94 were excluded for predefined reasons. These were: receipt of HDM without prior induction therapy (*n* = 23), substandard induction regimens (< triplet‐based; *n* = 39), low baseline difference in involved and uninvolved free light chains (dFLC < 20 mg/L; *n* = 8), and HDM or standard intensity treatment beyond 1 year after start of treatment (*n* = 24).

### 3.2 Clinical Characteristics

The final study cohort comprised 475 patients who met all inclusion criteria (Figure S1). Patients were relatively young (median age at diagnosis was 59.3 years, range 31–81), and ECOG scores were predominantly 0–1. Cardiac staging was available for 436 patients and showed stage 1 in 30.3%, stage 2 in 50.3%, and stage 3a in 19.5%, with no patients classified as stage 3b (Table S1). Regarding initial therapy, 78.3% of patients received bortezomib‐based induction, 18.7% received daratumumab‐based induction, and 2.1% received an IMiD‐based regimen without a proteasome inhibitor. Cytogenetic data demonstrated gain/amp1q in 19.4% out of 391 patients with assessable data and t(11;14) in 54.0% out of 426. High‐risk abnormalities, defined as del(17p), t(4;14), t(14;16), or t(14;20), were present in 12.4% (with 426 patients assessable). Patient characteristics between centers were mostly comparable, but there were significant differences in light chain isotype (*p* = 0.03), age (*p* = 0.003) and maintenance use (*p* = 0.05), without differences in TTP (*p* = 0.24) and OS (*p* = 0.88).

Landmark analyses including patients alive and progression free at 12 months after therapy initiation were performed to account for early non proportional hazards, avoid immortal time bias related to delayed HDM, and ensure completion of first line therapy and response assessment. A total of 109 patients received second‐line therapy or salvage HDM prior (Figure S2a). Nine patients died before reaching the landmark (Figure S2c), and six patients were lost to follow‐up during this period. After these exclusions, 351 patients remained eligible for landmarked analysis (Figure S1).

### 3.3 Variables Associated With Decision for HDM

In the resulting population comprising this study, 173 (49.3%) underwent HDM with autologous stem cell transplant and 178 (50.7%) received standard intensity first line regimen (Table 1). Compared with patients not receiving HDM, those proceeding to HDM were younger, had better ECOG performance status, and lower cardiac stage. Patients with high‐risk FISH results were more likely to proceed to HDM (79% vs. 40.7% of those with/without del17p and 51.9% vs. 39.4% with/without gain/amp1q went on to HDM). This was also true for patients with higher BMPC infiltration at diagnosis (58.5% vs. 41.5% of those with vs. without BMPC ≥ 10%). Patients with better responses were less likely to receive HDM: The proportion of those achieving ≥ VGPR was 76.5% in patients who did not proceed to HDM, whereas in those with HDM, it was 65.1%. Other baseline variables were broadly similar between groups. Of 164 patients in the HDM group with available melphalan dose data, 40 (24%) received < 200 mg/m^2^.

**TABLE 1:** Baseline characteristics after 12‐month landmark stratified by HDM.

Variable	All patients (*n* = 351)	No HDM (*n* = 178)	HDM (*n* = 173)	*p*
Age (y, range)	61 (31–72)	62 (39–72)	58 (31–63)	**< 0.001**
Female gender (%)	35.6	33.2	38.5	0.33
Date of diagnosis 2010–2015 (%)	23.6 (*n* = 80)	20.1 (*n* = 35)	27.3 (*n* = 45)	0.12
Date of diagnosis 2015–2020 (%)	49.0 (*n* = 166)	47.1 (*n* = 82)	50.9 (*n* = 84)	0.49
Date of diagnosis 2020–2024 (%)	27.4 (*n* = 91)	32.8 (*n* = 57)	21.8 (*n* = 36)	**0.02**
Lambda isotype (%)	80.0 (*n* = 350)	79.7 (*n* = 177)	80.4 (*n* = 173)	0.87
dFLC (mg/L; median, quartiles)	197 (77, 518; *n* = 345)	193 (75, 417; *n* = 175)	206 (87, 852; *n* = 170)	0.29
ECOG 0/1/2 (%)	52.0/40.4/7.6 (*n* = 275)	45.5/44.9/12.6 (*n* = 127)	60.1/36.5/3.4 (*n* = 148)	**0.001**
Cardiac stage (%; 1/2/3a/3b)	30.5/50.8/18.8/0 (*n* = 325)	26.6/47.9/25.5/0 (*n* = 169)	34.6/53.8/11.5/0	**0.008**
Renal stage (%; 1/2/3)	44.4/24.3/7.2 (*n* = 180)	52.8/40.4/6.7 (*n* = 89)	36.3/56.0/7.7 (*n* = 91)	0.08
GI involvement (%)	17.9 (*n* = 351)	18.0 (*n* = 178)	16.2 (*n* = 173)	0.66
Liver involvement (%)	9.7 (*n* = 351)	10.7 (*n* = 178)	8.7 (*n* = 173)	0.53
Nerve involvement (%)	15.7 (*n* = 351)	14.0 (*n* = 178)	17.3 (*n* = 173)	0.40
LDH (median, quartiles)	203 (168.5, 240.5; *n* = 117)	215 (169, 236.5; *n* = 62)	196 (160, 248; *n* = 55)	0.74
BMPC (%; median, quartiles)	10 (5.3, 20; *n* = 347)	10 (5.0, 15; *n* = 177)	13 (7, 20; *n* = 170)	**0.002**
Bortezomib‐based induction (%)	76.9 (*n* = 351)	49.7 (*n* = 178)	84.4 (*n* = 173)	**0.001**
IMiD‐based induction, no PI (%)	1.7 (*n* = 351)	0.6 (*n* = 178)	2.9 (*n* = 173)	**0.08**
Daratumumab‐based induction (%)	20.8 (*n* = 351)	29.2 (*n* = 178)	12.1 (*n* = 179)	**< 0.001**
Gain/amp1q (%)	20.7 (*n* = 290)	16.4 (*n* = 146)	25.0 (*n* = 144)	0.07
t(11;14) (%)	50.2 (*n* = 311)	50.3 (*n* = 157)	50.0 (*n* = 145)	0.96
Del17p, t(4;14), t(14;16), t(14;20) (%)	12.8 (*n* = 311)	10.9 (*n* = 156)	18.7 (*n* = 155)	0.06
Hyperdiploid karyotype (%)	15.2 (*n* = 310)	12.8 (*n* = 156)	17.5 (*n* = 154)	0.25
Other IGH translocations (%)	10.1 (*n* = 298)	9.7 (*n* = 144)	10.4 (*n* = 154)	0.85
≥ VGPR at 3 months (%)	60.0 (*n* = 270)	70.2 (*n* = 124)	51.4 (*n* = 146)	**0.002**
CR at 3 months (%)	25.9 (*n* = 270)	35.5 (*n* = 124)	17.8 (*n* = 146)	**0.001**
≥ VGPR ever/before HDM (%)	80.6 (*n* = 325)	90.4 (*n* = 167)	70.3 (*n* = 158)	**< 0.001**
CR ever/before HDM (%)	47.7 (*n* = 325)	56.3 (*n* = 167)	38.6 (*n* = 158)	**0.001**
CR/VGPR at 12‐month landmark (±1 month; %)	86.1/52.6 (*n* = 230)	87.7/50.9 (*n* = 106)	84.7/54.0 (*n* = 124)	0.51/0.64
Duration of therapy (without maintenance if received; months; median, quartiles)	5.0 (4.6, 6.5; *n* = 344)	6.0 (5.0; 8.2, *n* = 172)	4.7 (3.9, 7.6, *n* = 172)	0.31
Maintenance after induction/HDM (%)	27.1 (*n* = 336)	30.1 (*n* = 176)	23.8 (*n* = 160)	0.19
Duration of maintenance (months; median, quartiles)	18 (10.0, 22.0, *n* = 65)	17.0 (12.0, 21.0, *n* = 31)	18.5 (5.5, 23.5, *n* = 34)	0.60

*Note:* Bold values indicate statistical significance (*p* < 0.05).

Abbreviations: BMPC, bone marrow plasma cell percentage; Dara, daratumumab; dFLC, difference in free light chains; ECOG, eastern cooperative oncology group performance status; HDM, high dose melphalan; HR, high‐risk fluorescence in situ hybridization; IMiD, immunomodulatory drugs; LDH, lactate dehydrogenase; PI, proteasome inhibitors; R‐ISS, revised international staging system; VGPR, very good partial response.

### 3.4 Outcomes

Estimated median follow‐up for the landmarked HDM and non‐HDM groups was 83.6 and 75.6 months from diagnosis, respectively. The median time to progression (mTTP) of the overall cohort was 77.6 months. There were 77 (43.3%) patients without HDM and 77 (44.5%) patients after HDM who had progressed (*p* = 0.81). Of these, 33.5% and 36.5% fulfilled formal progression criteria, respectively; the others went on to second line due to the providers' conviction of meaningful serologic progression. Depth of response after first line therapy, defined at the 12‐month landmark, was similar with 84.7% and 54.0% in the HDM group versus 87.7% and 50.9% of patients in the non‐HDM group having reached a VGPR and CR, respectively. Median OS was not reached. OS after the 12‐month landmark was not statistically different stratified by HDM (Figure S2b) and Fine‐Gray sensitivity analysis showed death as an equally competing risk for both cohorts (*p* = 0.39). TTP after the 12‐month landmark was not different when stratified by HDM (Figure S2d).

To assess the impact of response state, cytogenetics, BMPC infiltration, and laboratory markers on outcomes, we analyzed univariable predictors of TTP. Elevated B2MG and LDH were not associated with altered TTP. dFLC > 180 mg/L was associated with shorter TTP in univariate analysis (HR 1.4). The remission states of VGPR and CR were associated with longer TTP (HR 0.49 and 0.29, respectively), whereas receipt of HDM and daratumumab was not.

Gain/amp1q was a very strong predictor of shorter TTP on univariate analysis (HR 2.15). BMPC > 20% was also associated with shorter TTP (HR 1.7), but high‐risk FISH and IGH translocations other than t(11;14) did not show worse outcomes (Table 2).

**TABLE 2:** Cox regression analyses for predictors of TTP after 12‐month landmark.

Risk factor for TTP	*n*/N	Univariable		Multivariable^a^	
		HR (95% CI)	*p*	HR (95% CI)	*p*
Best remission after standard intensity regimen/induction					
PR	50	Ref	Ref	Ref	Ref
VGPR	99	0.52 (0.30, 0.90)	**0.02**	0.49 (0.26, 0.92)	**0.03**
CR	185	0.29 (0.17, 0.49)	**< 0.001**	0.29 (0.16, 0.55)	**< 0.001**
B2mg > 5.5 mg/L	18/198	1.05 (0.45, 2.4)	0.91		
dFLC > 180 mg/L	183/351	1.4 (1.01, 1.9)	**0.04**	1.15 (0.76, 1.76)	0.51
LDH > Norm	23/122	0.68 (0.26, 1.76)	0.4		
BMPC %					
0%–5%	86	Ref	Ref	Ref	Ref
5%–20%	199	1.42 (0.94, 2.15)	0.1	1.57 (0.97, 2.55)	0.07
> 20%	62	1.70 (1.04, 2.80)	**0.04**	2.01 (1.10, 3.68)	**0.02**
+1q	60/299	2.15 (1.46, 3.16)	**< 0.001**	2.18 (1.44, 3.39)	**< 0.001**
del17p, t(4;14), t(14;16), t(14;20)^b^	46/311	1.40 (0.90, 2.18)	0.14		
t(11;14)	156/324	0.89 (0.64, 1.15)	0.51		
Receipt of HDM	173/351	0.88 (0.64, 1.20)	0.42	0.61 (0.40, 0.91)	**0.02**
Daratumumab containing regimen	73/351	0.59 (0.37, 1.03)	0.06	0.44 (0.22, 0.86)	**0.02**
Date of diagnosis					
2010–2015 (%)	80	Ref	0.13		
2015–2020 (%)	166	0.75 (0.52, 1.09)	0.38		
2020–2024 (%)	93	0.80 (0.48, 1.33)			
Receipt of any maintenance	102/351	0.75 (0.5, 1.12)	0.16		

*Note:* N/N refers to number of patients fulfilling criteria of those with available data. Bold values indicate statistical significance (*p* < 0.05).

Abbreviations: B2mg, β2‐microglobulin; BMPC, bone marrow plasma cell percentage; CI, confidence interval; CR, complete response; dFLC, difference in free light chains; HR, hazard ratio; LDH, lactate dehydrogenase; TP, time to progression; VGPR, very good partial response.

^a^ *n* = 272 patients with complete multivariable dataset, 154 events.

^b^ Assessed as one group due to low numbers in separate groups.

After multivariable adjustment, achievement of VGPR and CR after standard intensity therapy or induction predicted longer TTP (HR 0.49 and 0.29, respectively) and gain/amp1q remained a strong predictor of shorter TTP (HR 2.18). Daratumumab‐containing therapy and, importantly, receipt of HDM were also associated with longer TTP (HR 0.44 and 0.61, respectively, Table 2). ECOG, cardiac stage, and age that were different between HDM and no HDM group did not influence TTP. Inclusion of center or their differences (light chain isotype, maintenance use, age) as variables yielded similar significances. Sensitivity analysis using event‐free survival (EFS, Tables S2 and S3) including deaths or only formal ISA progression showed the same trends.

**FIGURE 1:**
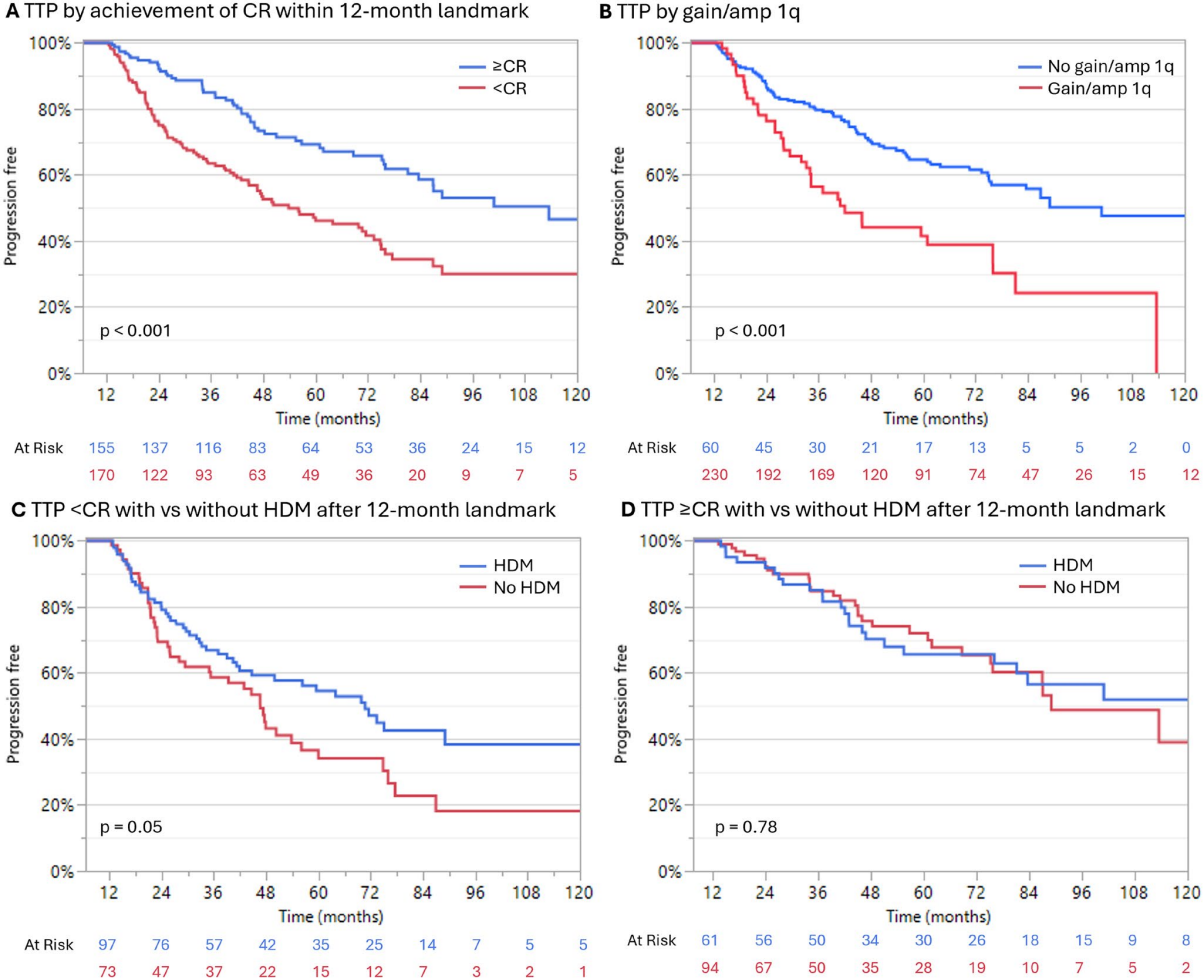
TTP after the 12‐month landmark according to hematologic response, cytogenetics, and receipt of HDM. (A) TTP stratified by achievement of CR within the first 12 months. (B) TTP according to gain or amplification of 1q. (C) TTP among patients who had not achieved CR within 12 months, stratified by receipt of HDM. (D) TTP among patients who achieved CR within 12 months, stratified by receipt of HDM. Patients who died or progressed within the first 12 months were excluded from the landmark analyses. *p* values were calculated using the log‐rank test. Numbers at risk are shown below each panel. [Color figure can be viewed at wileyonlinelibrary.com]

Kaplan–Meier analysis showed an mTTP of 113.5 vs. 53.8 months for patients having achieved CR vs. those who did not (Figure 1A) and a mTTP of 42.1 vs. 101.0 months for patients with vs. without gain/amp1q (Figure 1B).

### 3.5 Impact of HDM on Risk Factors for Earlier TTP

Patients with < CR after induction therapy that went on to HDM had significantly longer mTTP (21.0 vs. 5.7 months without HDM Figure 1C), a benefit not sustained if CR had been reached prior (53.4 vs. 52.7 months, respectively, Figure 1D).

These Kaplan–Meier findings were confirmed in univariable Cox proportional hazards models: achieving a deeper response after standard intensity regimen was associated with longer TTP if patients did not receive HDM (HR 0.16 in patients achieving a CR and a trend in patients with VGPR, HR 0.43, Table 3). However, reaching less than a CR or VGPR before HDM did not significantly impact time to progression (*p* = 0.55/0.36 with VGPR/CR before HDM, respectively). In Cox models including interaction terms, the effect of HDM on TTP did not differ significantly according to CR status or daratumumab exposure (*p* = 0.23 and *p* = 0.98, respectively). Interestingly, higher BMPC percentage at diagnosis was also a strong risk factor for earlier progression if patients did not receive HDM (HR 1.9 with 5%–20% BMPCs and 4.09 with > 20% BMPC), but in patients that received HDM, the risk for progression associated with higher BMPC infiltration was attenuated (HR 1.01 and 1.08, respectively).

**TABLE 3:** Univariable regression analysis for TTP after 12‐month landmark stratified by HDM.

Risk factor for shorter TTP	*n*/N	No HDM (*n* = 178)^a^		n/n	HDM (*n* = 173)^a^	
		HR (95% CI)	*p*		HR (95% CI)	*p*
Best remission after standard intensity regimen/induction						
PR	16	Ref	Ref	34	Ref	Ref
VGPR	57	0.43 (0.17, 1.09)	0.08	42	0.79 (0.37, 1.70)	0.55
CR	94	0.16 (0.06, 0.39)	**< 0.001**	91	0.70 (0.33, 1.49)	0.36
BMPC %						
0%–5%	50	Ref	Ref	36	Ref	Ref
5%–20%	106	1.90 (0.97, 3.73)	0.06	93	1.01 (0.44, 2.28)	0.99
> 20%	21	4.09 (1.6, 10.46)	**0.003**	41	1.08 (0.44, 2.60)	0.86
+1q	24/146	2.03 (1.1, 3.76)	**0.02**	36/144	2.46 (1.34, 4.51)	**0.004**
t(11;14)^b^	79/157	0.81 (0.46, 1.42)	0.46	77/154	1.09 (0.60, 2.01)	0.77
Melphalan dose < 200 mg/m^2^	—	—	—	40/164^c^	1.14 (0.67, 1.94)	0.63
Daratumumab containing regimen	52/178	0.61 (0.31, 1.21)	0.16	21/173	0.52 (0.19, 1.45)	0.21
Receipt of any maintenance	53/178	0.58 (0.32–1.05)	0.07	38/173	0.97 (0.56–1.67)	0.91

*Note:* N/N refers to number of patients fulfilling criteria of those with available data. Bold values indicate statistical significance (*p* < 0.05).

Abbreviations: BMPC, bone marrow plasma cell percentage; CI, confidence interval; FISH, fluorescence in situ hybridization; HDM, high dose melphalan; HR, hazard ratio; TTP, time to progression; VGPR, very good partial response.

^a^ There were 77 events in the no HDM cohort, and 77 events in the HDM cohort.

^b^ del17p, t(4;14), t(14;16), and t(14;20) were not assessed due to low numbers.

^c^ There were 22/40 events in the reduced HDM dose cohort, and 69/124 events in the cohort without melphalan dose reduction.

However, gain/amp1q remained an independent risk factor for earlier progression independent of HDM (mTTP 42.1/45.9 months, HR 2.46/2.03 with vs. without HDM, respectively). Formal interaction testing between HDM and gain/amp1q was not statistically significant (*p* = 0.44). A melphalan dose < 200 mg/m^2^ was not associated with inferior TTP (HR 1.14, *p* = 0.63). Sensitivity analyses using 9‐ and 18‐month landmarks yielded similar results (data not shown).

### 3.6 Predictors of Overall Survival

After the 12‐month landmark, 26 (14.6%) patients without HDM and 28 (16.2%) with HDM died (*p* = 0.68) within the observation period. Median OS was not reached; the estimated 5‐year OS after landmarking was 93.4% (95% CI 89.5%–97.4%) versus 91.2% (95% CI 86.5%–95.8%) in the HDM and non‐HDM groups, respectively. Age and depth of response were independently associated with OS (Table 4): having achieved CR after standard intensity therapy or induction independently predicted longer OS (HR 0.12). Other markers, including B2MG, dFLC, and LDH, showed no trend toward inferior OS. This was also true for BMPC percentage and gain/amp1q. Comparing OS in the HDM versus no HDM group, only higher age resulted in a slightly shorter OS in the HDM group. Among patients receiving HDM, 24% received reduced‐dose melphalan, with no difference in TTP or OS (Table 5).

**TABLE 4:** Cox regression analyses for predictors of OS after 12‐month landmark.

Risk factor for OS		Univariable		Multivariable^a^	
	*n*/N	HR (95% CI)	*p*	HR (95% CI)	*p*
Age (per year)	351	1.03 (1.01, 1.07)	**0.02**	1.04 (1.00, 1.08)	**0.03**
Best remission after standard intensity regimen/induction					
PR	50	Ref	Ref	Ref	Ref
VGPR	99	0.62 (0.27, 1.46)	0.27	0.61 (0.26, 1.45)	0.27
CR	185	0.41 (0.18, 0.94)	**0.04**	0.43 (0.19, 0.97)	**0.04**
B2mg > 5.5 mg/L	18/198	1.11 (0.66, 1.87)	0.67		
dFLC > 180 mg/L	183/351	0.99 (0.79, 1.24)	0.92		
LDH > Norm	23/122	1.06 (0.66, 1.70)	0.79		
BMPC %					
0%–5%	86	Ref	Ref		
5%–20%	199	1.04 (0.53, 2.04)	0.91		
> 20%	62	1.65 (0.75, 3.62)	0.21		
+1q	60/299	1.33 (0.64, 2.75)	0.44		
del17p, t(4;14), t(14;16), t(14;20)^b^	46/311	1.00 (0.44, 2.29)	0.99		
t(11;14)	156/324	1.37 (0.77, 2.44)	0.28		
Receipt of HDM	173/351	0.91 (0.54, 1.56)	0.74	0.89 (0.45, 1.74)	0.73
Daratumumab containing regimen	73/351	0.26 (0.04, 1.96)	0.19		
Date of diagnosis					
2010–2015 (%)	80	Ref	0.15		
2015–2020 (%)	166	0.64 (0.34, 1.18)	0.14		
2020–2024 (%)	93	0.32 (0.07, 1.45)			
Receipt of any maintenance	102/351	0.69 (0.31, 1.55)	0.38		

*Note:* N/N refers to number of patients fulfilling criteria of those with available data. Bold values indicate statistical significance (*p* < 0.05).

Abbreviations: 2mg, β2‐microglobulin; BMPC, bone marrow plasma cell percentage.CI, confidence interval; CR, complete response; dFLC, difference in free light chains; HR, hazard ratio; LDH, lactate dehydrogenase; TTP, time to progression; VGPR, very good partial response.

^a^ *n* = 325 patients with complete multivariable dataset, 48 events.

^b^ Assessed as one group due to low numbers in separate groups.

**TABLE 5:** Univariable regression analysis for OS after 12‐month landmark by HDM.

Risk factor for shorter OS	*n*/N	No HDM (*n* = 178)^a^		*n*/N	HDM (*n* = 173)^a^	
		HR (95% CI)	*p*		HR (95% CI)	*p*
Best remission after standard intensity regimen/induction						
PR	16	Ref	Ref	34	Ref	Ref
VGPR	57	0.14 (0.03, 0.74)	**0.02**	42	0.68 (0.22, 2.08)	0.5
CR	94	0.12 (0.02, 0.60)	**0.01**	91	0.31 (0.11, 0.90)	**0.03**
B2mg > 5.5 mg/L	10/89	1.37 (0.31, 6.16)	0.68	8/98	1.0 (0.99, 1.01)	0.99
dFLC > 180 mg/L	90/175	1.41 (0.63, 3.12)	0.40	77/170	1.77 (0.80, 3.92)	0.16
LDH > Norm	11/62	1.0 (0.99, 1.01)	0.99	12/55	1.0 (0.99, 1.01)	0.99
BMPC %						
0%–5%	50	Ref	Ref	36	Ref	Ref
5%–20%	106	0.85 (0.37, 1.97)	0.71	93	2.08 (0.34, 12.83)	0.43
> 20%	21	1.11 (0.38, 3.20)	0.85	41	—	
**Age at diagnosis**	178	1.03 (0.98, 1.08)	0.23	173	1.04 (1.0, 1.09)	**0.05**
+1q	24/146	1.55 (0.47, 5.0)	0.47	36/144	1.09 (0.43, 2.78)	0.84
t(11;14)^b^	79/157	1.78 (0.72, 4.36)	0.21	77/154	1.15 (0.54, 2.46)	0.71
Melphalan dose < 200 mg/m^2^	—	—	—	40/164	0.66 (0.23, 1.91)	0.44

*Note:* N/N refers to number of patients fulfilling criteria of those with available data. Impact of Daratumumab was not assessed due to insufficient events. Bold values indicate statistical significance (*p* < 0.05).

Abbreviations: B2mg, β2‐microglobulin; BMPC, bone marrow plasma cell percentage; CI, confidence interval; CR, complete response; dFLC, difference in free light chains; FISH, fluorescence in situ hybridization; HR, hazard ratio; LDH, lactate dehydrogenase; OS, overall survival; VGPR, very good partial response.

^a^ There were 26 events in the no HDM cohort, and 28 events in the HDM cohort.

^b^ del17p, t(4;14), t(14;16), and t(14;20) were not assessed due to low numbers.

## 4 Discussion

In this large multi‐center cohort of transplant‐eligible AL patients, patients with high BMPC in or hematologic response < CR after standard intensity regimen derived conditional benefit from HDM.

Earlier retrospective studies assessing outcomes and risk factors for progression and survival included both transplant‐eligible and ineligible patients. Given that HDM was considered the most effective therapy and was offered to all patients eligible, PFS and OS comparisons were highly in favor of HDM [10]. With more effective therapies in first and second line and the inclusion of daratumumab in first line, however, the role of HDM has been challenged and the incremental benefit of HDM has yet to be demonstrated.

We applied pre‐defined inclusion criteria to focus on transplant‐eligible patients and thus reduce selection bias in terms of treatment intensity. In the resulting cohort, the HDM cohort was still favored in age, performance status, and cardiac stage, but death was an equally distributed competing risk to TTP in both cohorts without differences in OS despite differences in baseline fitness and rates of cardiac involvement. However, the non‐HDM cohort had more favorable clonal disease features for TTP, with lower BMPC burden, fewer adverse cytogenetic features, and deeper responses to induction. Thus, interpretation of HDM effects on TTP and OS requires caution because overall treatment allocation remained non‐random.

Achieving a deep hematologic response prior to HDM [32, 33], after HDM [3, 5] or after consolidation with unsatisfactory response to HDM [34] is associated with better post‐transplant outcomes with longer PFS and OS. VGPR has been defined as a minimal requirement, but there is mounting evidence that CR [29, 35] or even minimal residual disease (MRD) negative status, either by bone marrow [36, 37] or peripheral blood mass spectrometry [38, 39] results in further improved outcomes. This was reflected in our data, where a response < VGPR after standard intensity regimen or induction resulted in a shorter mTTP of 40.4 vs. 83.6 months with ≥ VGPR. Our data add further evidence that CR should be achieved with treatment to optimize outcomes: TTP was even more favorable when patients achieved a CR after standard intensity regimen or induction (HR 0.29 with CR, 0.49 with VGPR compared to PR). This supports that when less than CR has been achieved, additional therapy is warranted [27, 40]. HDM can be a cost‐effective option in this at‐risk population not achieving deep responses after standard intensity regimen, and CR after HDM is a well‐established factor associated with better PFS and OS [1, 5, 41, 42, 43, 44, 45, 46]. Reduced melphalan dosing (< 200 mg/m^2^) was not associated with inferior TTP in univariate analysis, consistent with observations in multiple myeloma [47]. While attenuated dosing is established as effective in AL [42], prior studies have reported lower CR rates in upfront HDM without induction therapy [1].

Importantly, our data support the current real‐world strategy in which HDM is preferentially considered for patients with persistent clonal disease after induction: in patients with CR after induction therapy, TTP was 52.7 months compared with 53.4 months if they went on to HDM. In univariate regression analysis, the depth of remission prior to HDM did not predict TTP, as HDM compensates for poor remission [2]. The outcomes of this analysis thus support the current clinical approach that patients who attain < CR after standard intensity regimen benefit from consolidation with HDM, whereas patients in CR may choose to defer HDM [6, 48] or other therapies to deepen hematologic response without a trade‐off in OS, similar to approaches discussed for MM [49]. In the light of novel therapies with frequent deep hematologic responses, the role of HDM can be seen as a therapeutic option rather than pivotal therapy crucial for longer OS.

It is, however, important to note that responses beyond CR as defined by ISA such as MRD negativity have resulted in further improved outcomes, so that there may be benefit for some patients within those having achieved CR to receive further therapy [36, 37, 38, 39]. Importantly, formal interaction testing between receipt of HDM and achievement of CR after standard intensity therapy or induction was not statistically significant, indicating that some patients having achieved CR may still benefit from HDM, and MRD testing may be informative in this subgroup. However, sufficient MRD data were not available for this analysis.

We also confirm that patients with higher BMPC infiltration at diagnosis, parsed by 5% and 20% as proposed [19], have shorter TTP. Higher BMPC percentage at diagnosis is well described as an independent risk factor [19, 24]. In our patient cohort as well as in other international groups, this fact has resulted in a tendency to use HDM in this particular patient subset [15, 27, 50]. Interestingly, after parsing by receipt of HDM, we found that higher BMPC infiltration was not a risk factor for earlier TTP in the HDM group, suggesting an important role for patients with higher BMPC percentage beyond ISA defined hematologic response.

Multivariate analysis adjusted for gain/amp1q, which has been associated with inferior outcomes previously [22, 23, 24, 26] further confirmed it represents an independent risk factor for disease progression beyond its association with higher BMPC percentage: in our cohort, patients with vs. without gain/amp1q had a mTTP of 42.1 vs. 100.1 months without after the 12‐month landmark. Interestingly, patients with gain/amp1q did not benefit from HDM. We also did not detect a detrimental effect of t(11;14). Other cytogenetic risk markers such as del17p and translocations including the IGH locus were not associated with altered outcomes, but this analysis was underpowered due to low numbers as expected in AL cohorts [20]. High‐risk plasma cell risk stratifications requiring mutational analyses were not assessed due to low numbers. Deeper understanding of genome‐defined plasma cell trajectories may improve our knowledge on optimal upfront treatment choices in the future [51].

Importantly, slow or lack of deep hematologic response remained associated with inferior OS that was only partly mitigated by HDM: achieving < VGPR before transplant directly translated into a significantly inferior OS: 75% of patients that achieved VGPR were alive after 132.9 vs. after only 84.8 months if they had not. This once again highlights that achievement of rapid and deep responses is pivotal for AL patient survival. While HDM represents a widely available and cost‐effective option, it requires prior stem cell mobilization and collection. Novel off‐the‐shelf regimens, such as bispecific antibodies, are readily available and may therefore achieve faster hematologic control.

Only 20.8% of patients received daratumumab‐based induction therapy, but the biological principles we observe, that is, that BMPC burden and depth of response predict outcomes, and that HDM can overcome these risk factors, are likely treatment‐agnostic: while daratumumab‐based regimens achieve higher rates of deep response than historical triplets [13], they do not alter the proliferative capacity of residual clonal plasma cells [36, 37, 38]. Thus, the therapeutic strategy of using HDM to address inadequate disease control or more proliferative disease is relevant across treatment regimen.

Limitations of this study are inherent to retrospective study design, introducing a possible selection bias. Cytogenetic data were available in 60%–85% of patients depending on the abnormality, and particularly gain/amp1q, being associated with more proliferative phenotypes, may introduce bias. Despite transplant‐eligibility restriction and landmarking, decisions to proceed with HDM were clearly influenced by clinician judgment and center‐specific practice patterns. Importantly, induction response still influenced HDM allocation, whereas plasma cell risk factors were more prevalent in the HDM group. Induction regimens were not uniform, and differences in treatment intensity or tolerability may have contributed to the decision for or against HDM. These imbalances can influence the magnitude of treatment associations. A common problem in AL is that second‐line therapies were often initiated based on provider‐determined serologic progression, introducing potential bias in TTP analyses. However, EFS using formal ISA progression criteria showed the same trends. A prospective trial to assess the impact of HDM is recruiting [52].

## 5 Conclusion

HDM provided a clear progression benefit for individuals with BMPC infiltration > 20% or hematologic response < CR after standard‐intensity induction, while in patients achieving a CR, no consistent advantage was detected. Gain/amp1q remained a strong predictor of earlier progression regardless of treatment, and HDM did not mitigate its impact. Overall, HDM should be viewed as an additional option for patients with persistent disease activity after standard intensity regimen or higher proliferative burden at diagnosis in transplant‐eligible patients.

## Author Contributions

Steinhardt M.J. collected the data, contributed data, designed the analysis, analyzed and interpreted data, performed statistical analysis, and wrote the paper. Hegenbart U collected the data, contributed to data, analyzed, and interpreted data. Hellou T collected the data, contributed to the data, analyzed and interpreted the data. Oubari S collected the data, contributed to the data, analyzed, and interpreted data. Roussel M collected the data, contributed to the data, analyzed, and interpreted the data. Schwotzer R collected the data, contributed to data, analyzed, and interpreted data. Buadi F contributed data, analyzed, and interpreted data. Dingli D contributed data, analyzed, and interpreted data. Binder M contributed data, analyzed, and interpreted data. Zanwar S.S. contributed data, analyzed, and interpreted data. Kourelis T contributed data, analyzed, and interpreted data. Rieger M.J. contributed data, analyzed, and interpreted data. Morbach C contributed data, analyzed, and interpreted data. Cejka V collected the data, contributed to data, analyzed and interpreted data. Gerber B contributed data, analyzed, and interpreted data. Störk S contributed data, analyzed, and interpreted data. Einsele H contributed data, analyzed, and interpreted data. Kumar S contributed data, analyzed, and interpreted data. Gertz M.A. contributed data, analyzed, and interpreted data. Carpinteiro A collected the data, contributed to the data, analyzed, and interpreted data. Muchtar E collected the data, contributed data, analyzed, and interpreted data. Kortum K.M. contributed data, analyzed, and interpreted data. Dispenzieri A contributed data, designed the analysis, analyzed and interpreted data, performed statistical analysis, and wrote the paper. Schönland S contributed data, designed the analysis, analyzed and interpreted data, performed statistical analysis, and wrote the paper.

## Conflicts of Interest

Steinhardt M.J. received travel funds from and consultancy for Johnson&Johnson and Alexion and received research funding from Bayer outside of the submiwotzer R received advisory/consulting fees fromtted work. Oubari S received travel and congress funds from Johnson&Johnson and Alexion. Sch Alnylam, Astrazeneca, Johnson&Johnson; financial support for ‘amyloidosis center of excellence’ from Alnylam, AstraZeneca, SOBI, Pfizer, Bayer; travel grant from Johnson&Johnson. Dingli D has received honoraria and serves in a consulting role for Sorrento, Alexion Pharmaceuticals, Apellis Pharmaceuticals, Argenx, Bristol Myers Squibb Foundation, Johnson&Johnson, Novartis, Regeneron, Sanofi, and receives research funding from K36 Therapeutics. Kourelis T receives research funding from Novartis and Pfizer. Gerber B has received grant support from Pfizer, and honoraria from Johnson&Johnson, Sanofi, and Abbvie. Rieger M.J. received travel grants from Amgen and serves in a consulting role for Amgen and Sobi. Kumar S.K. serves in a consulting role for Takeda, Janssen Oncology, Genentech/Roche, Abbvie, Bristol‐Myers Squibb/Celgene, Pfizer, Regeneron, Sanofi, K36 Therapeutics, has received travel grants from Abbvie, Pfizer, Janssen, Beigene, and receives research funding from Takeda, Abbvie, Novartis, Sanofi, Janssen Oncology, MedImmune/AstraZeneca, Roche/Genentech, CARsgen Therapeutics, Allogene Therapeutics, GlaxoSmithKline, Regeneron, Bristol‐Myers Squibb/Celgene, Merck, and Oncopeptides (independent review committee participation). Gertz M.A. serves in a consulting role for Prothena and Bristol‐Myers Squibb/Sanofi, has received travel grants from Prothena, Celgene, Novartis, and has received honoraria from Celgene, Med Learning Group, Research to Practice, Prothena, Apellis Pharmaceuticals, Amgen, Abbvie, Akcea Therapeutics, Sanofi, Telix Pharmaceuticals, Janssen Oncology, Juno/Celgene, and Alnylam. Carpinteiro A received consulting and/or lecture fees from Alexion, Alnylam, Johnson&Johnson, Pfizer, and Sanofi, and travel and congress participation grants from Johnson&Johnson. Muchtar E serves in a consulting role for Protego. Kortum K.M. reports honoraria and research support from AbbVie, BMS, GSK, Janssen, Menarini, Novartis, Pfizer, Regeneron, Roche, Sanofi, and Skyline Dx. Dispenzieri A serves in a consulting role for Janssen Research & Development, has received travel grants from Pfizer, Janssen Oncology, Prothena, and receives research funding from Celgene, Janssen Oncology, Pfizer, Takeda, Alnylam, and Prothena. Hegenbart U, Roussel M, Hellou T, Buadi F, Binder M, Zanwar S.S., Morbach C, Cejka V, Störk S, Einsele H and Schönland S report no relevant conflicts of interest.

## Supporting information


**Table S1:** Baseline characteristics of non‐landmarked population.
**Table S2:** Cox regression analyses for predictors of EFS.
**Table S3:** Cox regression analysis for EFS stratified by receipt of HDM a there were 90 events in the no HDM cohort, and 84 events in the HDM cohort.
**Figure S1:** Flowchart of cohort selection.
**Figure S2:** Overall survival and time to progression before and after 12‐month landmark analysis.

## Data Availability

The data that support the findings of this study are available from the corresponding author upon reasonable request.
