# Supplementary material for: Determinants of Long‐Term Benefit From High Dose Melphalan With Autologous Stem Cell Transplant in AL Amyloidosis
Source: Am J Hematol. 2026 Jun 7;101(8):1903–14. doi: 10.1002/ajh.70371 (PMC13331647; doi:10.1002/ajh.70371)

| **Variable** | **All patients (n=475)** | **No HDM n=272** | **HDM n=203** | **P value** |
| --- | --- | --- | --- | --- |
| Age (y, range) | 59.3 (31-81) | 62.0 (32-81, n=272) | 58.0 (31-77, n=203) | **<0.001** |
| Female gender (%) | 34.3 | 33.5 (n=272) | 35.5 (n=203) | 0.65 |
| Date of diagnosis 2010-2015 (%) | 22.1 (105/475) | 17.2 (47/272) | 28.7 (58/203) | 0.37 |
| Date of diagnosis 2015-2020 (%) | 50.6 (241/475) | 50.8 (138/272) | 50.5 (103/203) | **0.01** |
| Date of diagnosis 2020-2024 (%) | 27.3 (129/475) | 32.0 (87/272) | 20.8 (42/203) | **<0.001** |
| Lambda isotype (%) | 77.8 (n=474) | 77.5 (n=271) | 78.3 (n=203) | 0.82 |
| dFLC (mg/L; median, quartiles) | 197 (84; 512) | 190 (83; 423; n=266) | 212 (89; 580; n=199) | 0.18 |
| ECOG 0/1/2 (%) | 47.7/44.2/8.1 (n=369) | 39.3/50.0/11.7 (n=196) | 57.2/38.7/4.1 (n=173) | **<0.001** |
| Cardiac stage (%; 1/2/3a/3b) | 30.3/50.3/19.5/0 (n=436) | 27.7/46.9/25.4/0 (n=256) | 33.9/55.0/11.1/0 (n=180) | **0.002** |
| Renal stage (%; 1/2/3) | 42.4/48.4/9.2 (n=342) | 46.6/44.6/8.8 (n=193) | 43.0/49.0/8.0 (n=149) | 0.72 |
| GI involvement (%) | 16.8 (n=475) | 16.9 (n=272) | 16.8 (n=203) | 0.59 |
| Liver involvement (%) | 11.4 (n=475) | 13.2 (n=272) | 8.9 (n=203) | 0.13 |
| Nerve involvement (%) | 14.5 (n=475) | 13.2 (n=272) | 16.3 (n=203) | 0.36 |
| LDH (median, quartiles) | 207 (168.75, 243.75; n=166) | 213 (172, 239.5; n=101) | 198 (159.5, 248; n=65) | 0.49 |
| BMPC (%; median, quartiles) | 10 (5; 20; n=469) | 10 (5.0; 15.5; n=269) | 13 (7; 20; n=200) | **0.001** |
| Bortezomib-based induction (%) | 78.3 (n=475) | 73.5 (n=272) | 84.7 (n=203) | **0.003** |
| IMiD-based induction, no PI (%) | 2.1 (n=475) | 1.1 (n=272) | 3.5 (n=203) | **0.08** |
| Daratumumab-based induction (%) | 18.7 (n=475) | 22.6 (n=272) | 12.9 (n=203) | **0.05** |
| Gain/amp1q (%) | 19.4 (n=391) | 16.7 (n=227) | 25.0 (n=164) | **0.04** |
| t(11;14) (%) | 54.0 (n=426) | 57.3 (n=246) | 49.4 (n=180) | 0.11 |
| Del 17p, t(4;14), t(14;16), t(14;20) (%) | 12.4 (n=426) | 8.6 (n=245) | 17.7 (n=181) | **0.005** |
| Hyperdiploid karyotype (%) | 15.3 (n=425) | 12.7 (n=245) | 18.9 (n=180) | 0.08 |
| Other IGH translocations (%) | 9.4 (n=413) | 8.6 (n=233) | 10.6 (n=180) | 0.50 |
| ≥ VGPR at 3 months (%) | 52.3 (n=369) | 56.8 (n=199) | 17.1 (n=170) | 0.06 |
| CR at 3 months HDM (%) | 21.1 (n=369) | 25.1 (n=199) | 16.5 (n=170) | **0.04** |
| ≥ VGPR ever/before HDM (%) | 71.7 (n=441) | 76.5 (n=255) | 65.1 (n=186) | **0.009** |
| CR ever/before HDM (%) | 39.5 (n=441) | 42.4 (n=255) | 35.5 (n=186) | 0.14 |
| Duration of therapy (without maintenance if received; months; median, quartiles) | 5.1 (4.4; 5.7, n=457) | 5.5 (3.6; 6.6, n=261) | 4.9 (4.0; 6.3, n=196) | 0.48 |
| Maintenance after induction/HDM (%) | 22.8 (n=448) | 23.4 (n=261) | 21.9 (n=187) | 0.72 |
| Duration of maintenance (months; median, quartiles) | 18 (10.0, 22.0, n= 69) | 17.0 (11.5, 21.5, n=33) | 21.5 (12.125, 24.25, n=36) | 0.53 |

**Table S1: Baseline characteristics of non-landmarked population.**

HDM, high dose melphalan; ECOG, Eastern Cooperative Oncology Group performance status; dFLC, difference in free light chains; IMiD, immunomodulatory drugs; LDH, lactate dehydrogenase; R-ISS, Revised International Staging System; BMPC, bone marrow plasma cell percentage; HR, high-risk fluorescence in situ hybridization; PI, proteasome inhibitors; VGPR, very good partial response; HDM, high dose melphalan.

| **Risk factor for EFS** | **n/N** | **Univariable** | | **Multivariable^a^** | |
| --- | --- | --- | --- | --- | --- |
|  |  | HR (95% CI) | p Value | HR (95% CI) | p Value |
| **Best remission after standard intensity regimen/induction**  PR  VGPR  CR | 50  99  185 | Ref  0.82 (0.50-1.32)  0.51 (0.32-0.01) | Ref  0.41  **0.004** | Ref  0.72 (0.41-1.24)  0.40 (0.23-0.70) | Ref  0.24  **0.001** |
| **B2mg >5.5 mg/L** | 18/198 | 1.01 (0.46-2.20) | 0.98 |  |  |
| **dFLC >180 mg/L** | 183/351 | 1.31 (0.97-1.18) | 0.08 |  |  |
| **LDH > Norm** | 23/122 | 0.58 (0.21-1.38) | 0.2 | 1.07 (0.72-1.58) | 0.75 |
| **BMPC %**  0-5%  5-20%  >20% | 86  199  62 | Ref  1.26 (0.86-1.83)  1.15 (0.91-2.29) | Ref  0.23  0.12 | Ref  1.44 (0.91-2.28)  1.83 (1.02-3.30) | Ref  0.12  **0.04** |
| **+1q** | 60/299 | 2.02 (1.39-2.95) | **<0.001** | 2.23 (1.47-3.38) | **<0.001** |
| **del17p, t(4;14), t(14;16), t(14;20)^b^** | 46/311 | 1.26 (0.82-1.93) | 0.29 |  |  |
| **t(11;14)** | 156/324 | 0.92 (0.67-1.27) | 0.61 |  |  |
| **Receipt of HDM** | 173/351 | 0.81 (0.60-1.09) | 0.16 | 0.78 (0.45-1.02) | 0.06 |
| **Daratumumab containing regimen** | 73/351 | 0.45 (0.25-0.82) | **0.008** | 0.43 (0.21-0.91) | **0.03** |
| **Date of diagnosis**  2010-2015 (%)  2015-2020 (%)  2020-2024 (%) | 80  166  93 | Ref  0.75 (0.53-1.05)  0.70 (0.42-1.15) | Ref  0.1  0.16 |  |  |
| **Receipt of any maintenance** | 102/351 | 0.66 (0.44-0.98) | **0.04** | 0.73 (0.45-1.19) | **0.73** |

**Table S2:** **Cox regression analyses for predictors of EFS.**

a n=258 patients with complete multivariable dataset, 127 events.

b assessed as one group due to low numbers in separate groups.

TTP, time to progression; HR, hazard ratio; CI, confidence interval; VGPR, very good partial response; CR, complete response; B2mg, β2-microglobulin; dFLC, difference in free light chains; LDH, lactate dehydrogenase; BMPC, bone marrow plasma cell percentage; N/N refers to number of patients fulfilling criteria of those with available data.

| **Risk factor for shorter EFS** | **n/N** | **No HDM (n=178)^a^** | | **n/n** | **HDM (n=173)^a^** | |
| --- | --- | --- | --- | --- | --- | --- |
|  |  | HR (95% CI) | p Value |  | HR (95% CI) | p Value |
| **Best remission after standard intensity regimen/induction**  PR  VGPR  CR | 16  57  94 | Ref  0.41 (0.19-0.88)  0.19 (0.09-0.41) | Ref  **0.23**  **<0.001** | 34  42  91 | Ref  0.72 (0.37-1.40)  0.63 (0.34-1.18) | Ref  0.34  0.15 |
| **BMPC %**  0-5%  5-20%  >20% | 50  106  21 | Ref  1.72 (1.02-2.89)  2.80 (1.34-5.84) | Ref  **0.04**  **0.006** | 36  93  41 | Ref  0.93 (0.53-1.62)  1.07 (0.58-1.99) | Ref  0.80  0.83 |
| +1q | 24/146 | 1.77 (1.0-3.15) | **0.05** | 36/144 | 2.20 (1.32-3.66) | **0.003** |
| t(11;14) ^b^ | 79/157 | 0.83 (0.53-1.31) | 0.43 | 77/154 | 1.03 (0.65-1.61) | 0.91 |
| Melphalan dose <200 mg/m^2^ | - | - | - | 40/164 | 0.92 (0.54-1.56) | 0.75 |
| Daratumumab containing regimen | 52/178 | 0.51 (0.26-1.0) | **0.05** | 21/173 | 0.26 (0.06-1.04) | 0.06 |
| Receipt of any maintenance | 53/178 | 0.49 (0.27-0.88) | **0.02** | 38/173 | 0.89 (0.52-1.53) | 0.68 |

**Table S3: Cox regression analysis for EFS stratified by receipt of HDM**
a there were 90 events in the no HDM cohort, and 84 events in the HDM cohort.

b del17p, t(4;14), t(14;16), and t(14;20) were not assessed due to low numbers.

c there were 22/40 events in the reduced HDM dose cohort, and 69/124 events in the cohort without melphalan dose reduction.

TTP, time to progression; HR, hazard ratio; CI, confidence interval; VGPR, very good partial response; FISH, fluorescence in situ hybridization; BMPC, bone marrow plasma cell percentage; HDM, high dose melphalan. N/N refers to number of patients fulfilling criteria of those with available data.

# Figure legends

**Figure S1:** **Flowchart of cohort selection.**

HDM, high dose melphalan; dFLC, difference in free light chains; EHA-ISA, European Hematology Association-International Society of Amyloidosis.

**Figure S2. Overall survival and time to progression before and after 12-month landmark analysis.**

(A) Kaplan-Meier estimate of overall survival (OS) for the entire cohort before landmarking. (B) OS after a 12-month landmark, stratified by receipt of high-dose melphalan (HDM) versus no HDM. (C) Kaplan-Meier estimate of time to progression (TTP) before landmarking. (D) TTP after a 12-month landmark, stratified by HDM versus no HDM.

Patients who died or progressed within the first 12 months were excluded from the landmark analyses. P values were calculated using the log-rank test. Numbers at risk are shown below each panel.

# Figures

**Figure S1:** **Flowchart of cohort selection.**


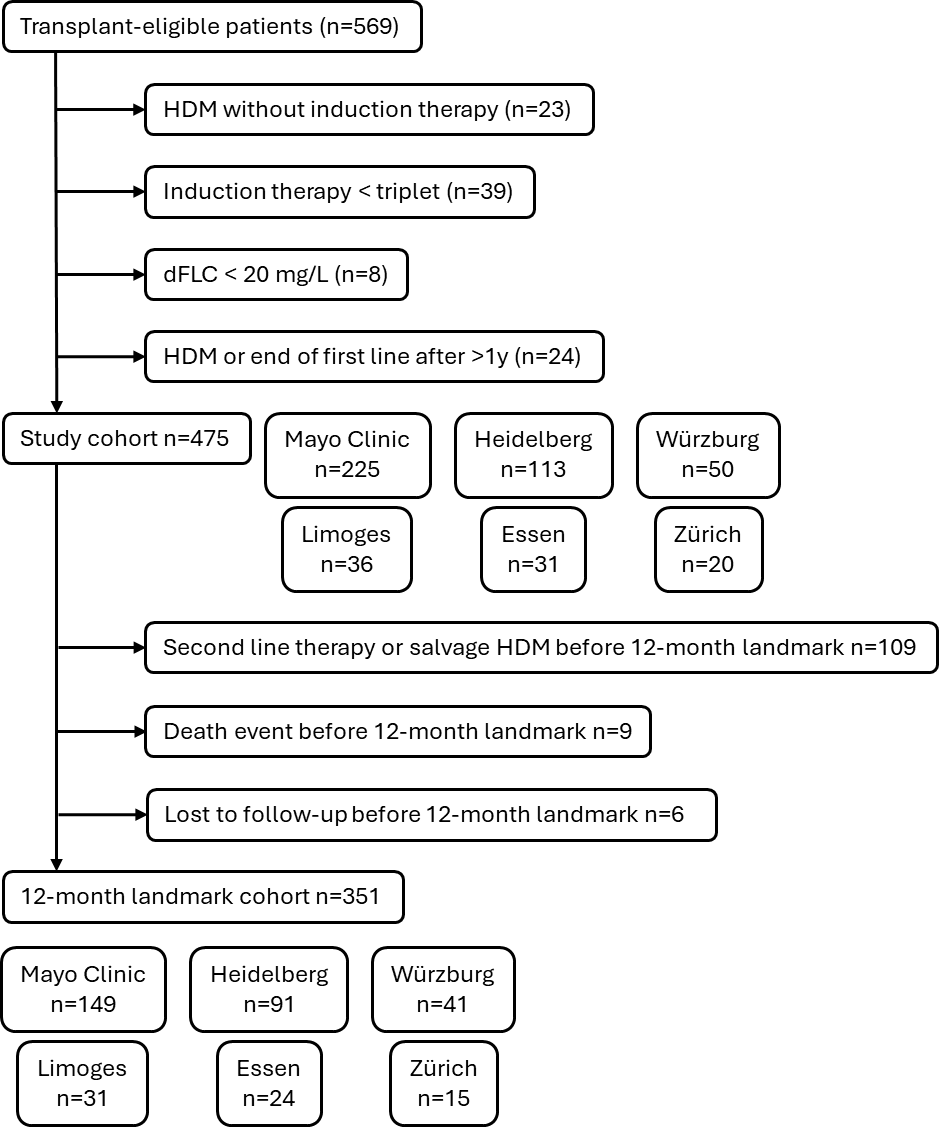


**Figure S2:** **Overall survival and time to progression before and after 12-month landmark analysis**


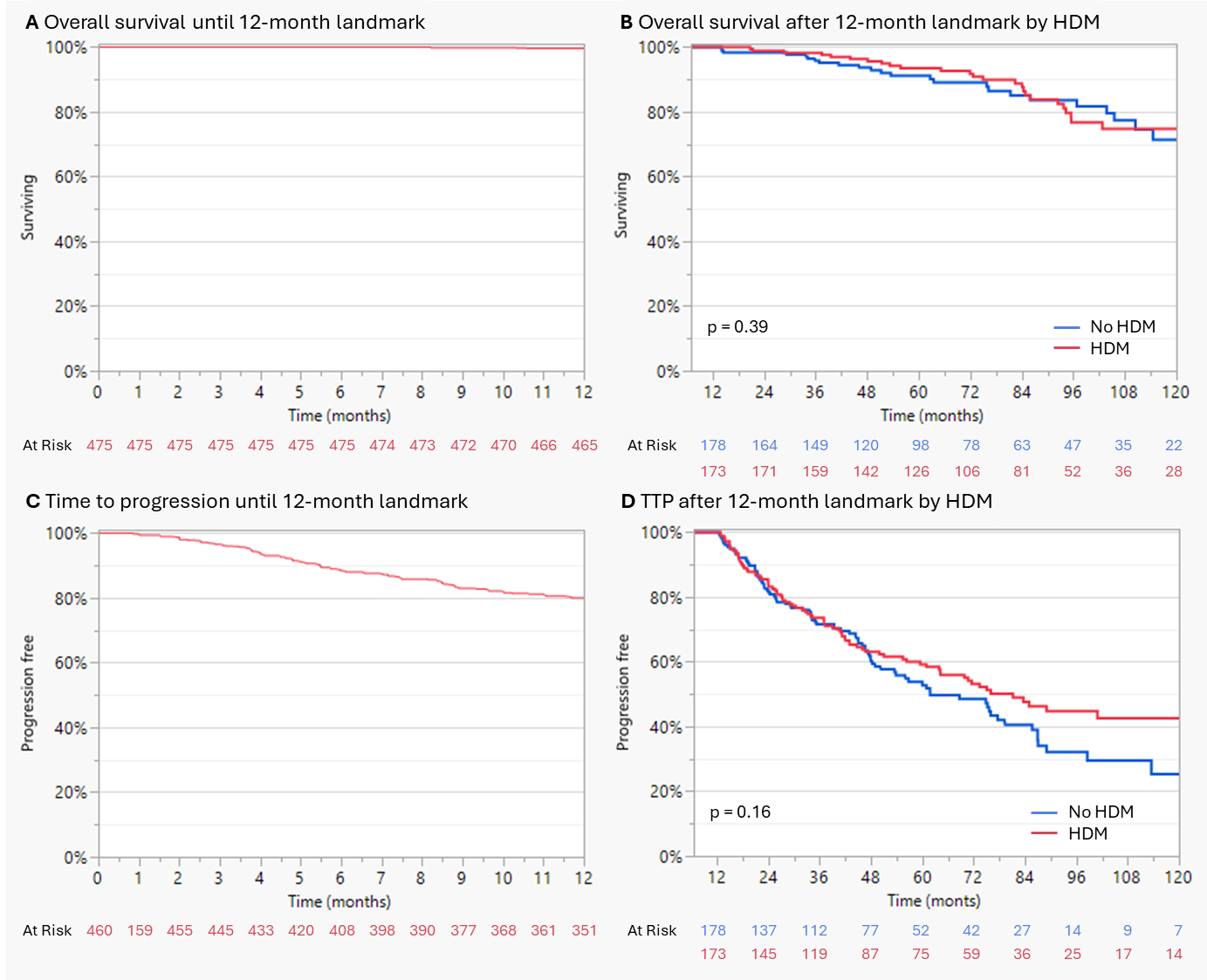

Supplement: Supplementary file 1 — Table S1: Baseline characteristics of non‐landmarked population. Table S2: Cox regression analyses for predictors of EFS. Table S3: Cox regression analysis for EFS stratified by receipt of HDM a there were 90 events in the no HDM cohort, and 84 events in the HDM cohort. Figure S1: Flowchart of cohort selection. Figure S2: Overall survival and time to progression before and after 12‐month landmark analysis. [file AJH-101-1903-s001.docx]
